# Antibacterial Activity of pH-Dependent Biosynthesized Silver Nanoparticles against Clinical Pathogen

**DOI:** 10.1155/2014/725165

**Published:** 2014-05-21

**Authors:** Kethirabalan Chitra, Gurusamy Annadurai

**Affiliations:** Environmental Nanotechnology Division, Sri Paramakalyani Centre for Environmental Sciences, Manonmaniam Sundaranar University, Alwarkurichi, Tamilnadu 627412, India

## Abstract

Simple, nontoxic, environmental friendly method is employed for the production of silver nanoparticles. In this study the synthesized nanoparticles UV absorption band occurred at 400 nm because of the surface Plasmon resonance of silver nanoparticles. The pH of the medium plays important role in the synthesis of control shaped and sized nanoparticles. The colour intensity of the aqueous solution varied with pH. In this study, at pH 9, the colour of the aqueous solution was dark brown, whereas in pH 5 the colour was yellowish brown; the colour difference in the aqueous solution occurred due to the higher production of silver nanoparticles. The antibacterial activity of biosynthesized silver nanoparticles was carried out against *E. coli*. The silver nanoparticles synthesized at pH 9 showed maximum antibacterial activity at 50 **μ**L.

## 1. Introduction

Nanoscience and nanotechnology is an emerging field, which involves in the synthesis, application of nanoscale materials, and structures usually in the range of 1 to 100 nm [1]. Due to the optical, electronic, magnetic, and chemical properties and their possible applications in subsequent technology development, nanoparticles synthesis has received considerable attention in recent years [2]. Metal nanoparticles are having considerable interest in the fast-developing area of nanotechnology because of its applications [3]. Currently various types of metal inorganic nanoparticles zinc, titanium, magnesium, copper, gold, alginate, and silver have been synthesized using various techniques [4].

Biological synthesis of nanoparticles is an alternative method of chemical and physical methods; various organisms are used for nanoparticles synthesis, because of its effectiveness and flexible biological factors [5, 6]. A major aim of research in nanotechnology is a synthesis of greener nanomaterials and the development of swift and steadfast experimental protocols for the synthesis of green nanomaterials includes a range of size, chemical compositions, high monodispersity and large scale production which are the key features of nanotechnology [7]. There is a great need to develop clean, nontoxic chemicals and environmentally benign solvents and renewable materials mediated synthesis method; thus a biological mediated synthesis of nanoparticles has received significant consideration in the last decade [8]. Both unicellular and multicellular organisms produce inorganic materials by intracellular or extracellular method. Magnetotactic bacteria, diatoms, and S-layer bacteria are good examples of microorganisms producing inorganic materials [9].

Metallic nanoparticles have been used in biosensing, media recording, optics, catalysis, and environmental remediation [10]. Traditionally silver has been used in customary medicine to gastronomic items because of their disinfecting effect [11]. Because of their unique optoelectronic and physicochemical properties, silver nanoparticles have attracted remarkable attention. Due to their distinctive properties such as good electrical conductivity, chemical stability, and catalytic and antibacterial activities, the silver nanoparticles are gaining more interest and most widely used [12]. The noxious nature of silver nanoparticles against various microorganisms has been well known; because of their antibacterial properties, silver nanoparticles are being used in the formulation of dental resin composites and ion exchange fibers and in coatings for medical devices [13, 14].

In this study, the silver nanoparticles were synthesized by an extracellular synthesis process using* Bacillus brevis *cell culture and then the effect of pH on the synthesis of silver nanoparticles was examined by changing the pH of the aqueous cell filtrate with 0.1 N sodium hydroxide and hydrochloric acid. The synthesized nanoparticles were characterized; the antibacterial activity of silver nanoparticles was examined against* E. coli*.

## 2. Experimental

### 2.1. Materials

Silver nitrate, Nutrient agar, Nutrient broth, Luria Bertani medium, Sodium chloride, and Hydrochloric acid were obtained from Himedia Pvt. Ltd., India.* E. coli* was purchased from Microlab, Arcot, Tamilnadu. The bacteria were isolated from pond water and identified as* Bacillus brevis* using Bergey's manual.

### 2.2. Extracellular Synthesis of Silver Nanoparticles

The fresh* Bacillus brevis* culture was inoculated in nutrient broth and the flask was incubated in orbital shaker at room temperature for 24 hrs. After 24 hrs the culture was centrifuged at 10,000 rpm for 10 minutes, and then the obtained supernatant was collected in a conical flask. 1 mM of silver nitrate was added to the culture supernatant to the synthesis of the silver nanoparticles, and then the flask was incubated at room temperature in orbital shaker for 48 hrs. The UV absorption spectrophotometer reading was taken at different time intervals to monitor the synthesis of silver nanoparticles extracellularly.

### 2.3. Effect of pH on the Extracellular Synthesis of Silver Nanoparticles

The influences of pH on the extracellular synthesis of silver nanoparticles were carried out by changing the pH of the bacterial extracellular aqueous media. The different pH was taken (5 and 9) to examine the effect of pH on the synthesis of silver nanoparticles using* Bacillus*. The pH of the extracellular aqueous media was changed using 0.1 N Hydrochloric acid and 0.1 N Sodium hydroxide. UV-spectrophotometer was used to take the absorption at 24 hrs of incubation.

### 2.4. Characterization of Biosynthesized Silver Nanoparticles

The silver nanoparticles were synthesized using the above-mentioned process and then the air dried sample was used to characterization technique. The UV (Perkin Elmer) absorbance spectra were taken at various time intervals at different wavelength. Powder X-ray diffractometer (Bruker D8 Advance uses CuK*α* radiation, at the 40 kev in the range of 10–80) was used to analyze the nature of the nanoparticles. Scanning electron microscope was used to identify the morphology of the synthesized silver nanoparticles. EDAX was used to show the element of the nanoparticles. The functional groups of biologically synthesized dried nanoparticles were observed using Fourier Transform Infrared Spectrometer (Thermo Nicolet Model: 6700). The sample mixed with KBr and then pressed into thin pellet. Infrared spectra were measured at the wavelength in the range of 400–4000 cm^−1^.

### 2.5. Antibacterial Activity of Silver Nanoparticles

The antibacterial activity of biosynthesized silver nanoparticles was carried out against* Escherichia coli. *Various concentrations (10 *μ*L, 20 *μ*L, 30 *μ*L, 40 *μ*L, and 50 *μ*L) of silver nanoparticles (synthesized using different pH (5 and 9) and original pH (7.2)) were used for examining the antibacterial activity of silver nanoparticles. The well-diffusion method was used to determine the antibacterial activity of silver nanoparticles; the well was formed in the medium using needle; then the colloidal silver nanoparticles were pipette out into the wells, and then the plates were incubated at 37°C for 24 hrs. After 24 hrs of incubation, the plates were observed for the zone of inhibition.

## 3. Results and Discussion 

Several physical and chemical methods have been used for the synthesis of metallic nanoparticles; however, there is a need to develop simple and ecofriendly method to synthesis the metallic nanoparticles [15]. As a result of the growing success and simple process for the nanoparticles formation, the biological organisms in this field are swiftly gaining importance [16]. Silver nanoparticles have attractive physicochemical properties; therefore, silver nanoparticles play a profound role in the area of biology and medicine [17]. In the present study, an ecological cost effective method is employed for the extracellular synthesis of silver nanoparticles using* Bacillus brevis *cell filtrate.

**Figure 1:**
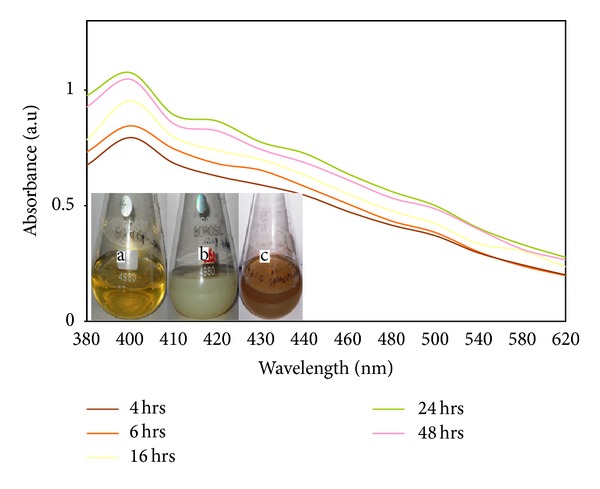
UV-spectrophotometer absorption of silver nanoparticles synthesized using* Bacillus brevis*, inset (a) bacterial culture before the addition of silver nitrate, (b) after addition of silver nitrate, and (c) after 24 hrs of reaction.

**Figure 2:**
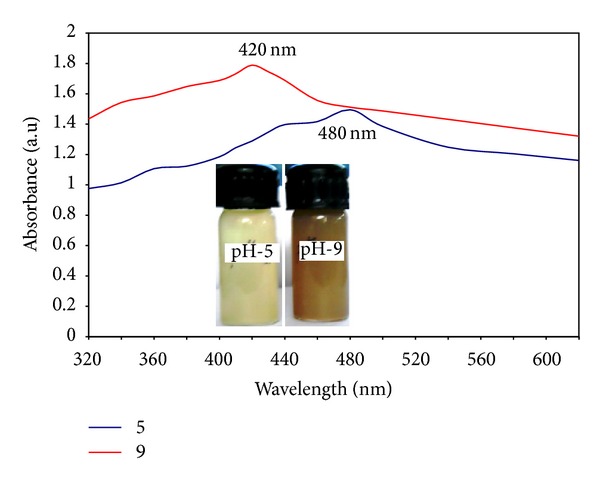
UV-spectrophotometer absorption of the effect of pH on the synthesis of silver nanoparticles. Inset shows that the colour variation at pH 5 and the synthesis of silver nanoparticles is low and at pH 9 the production of silver nanoparticles is high.

**Figure 3:**
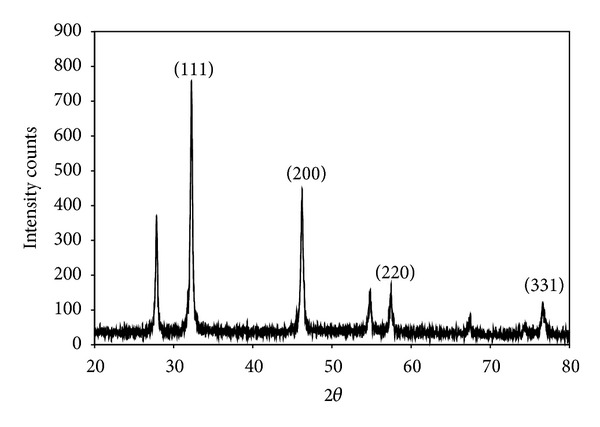
X-ray diffractometer of silver nanoparticles synthesized using* Bacillus brevis*.

The preliminary confirmation for the formation of silver nanoparticles was the visual observation of colour change of the aqueous solution of bacterial culture. Before (a) the addition of silver nitrate the culture was in yellow colour and after (b) addition of silver nitrate, the extracellular culture colour was changed to white precipitate and at 24 hrs (c) of reaction, the colour of the solution was changed to brown (Figure 1 inset). Kalimuthu et al. [18] synthesized silver nanocrystals using* Bacillus licheniformis*; they obtained the similar colour changes during the formation of silver nanoparticles. The UV absorption spectral studies were carried out to confirm the formation of silver nanoparticles using* Bacillus brevis*. Figure 1 shows UV absorption spectrum; the peak is found at 400 nm and the maximum absorption peak occurred at 24 hrs. Because of the excitation of surface Plasmon resonance, the colour change occurred after the addition of silver nitrate in the extracellular aqueous medium; it indicates the formation of silver nanoparticles [12]. Prakash et al. [19] synthesized silver nanoparticles using* Bacillus megaterium*; they obtained the maximum absorption peak at 435 nm and they stated the band occurred due to the surface Plasmon resonance of silver nanoparticles.  Figure 2 shows the effect of pH on the synthesis of silver nanoparticles. At pH 9, the maximum production of silver nanoparticles occurred. The absorption peak occurred at 420 nm and 460 nm for pH 9 and pH 5, respectively. The inset of Figure 2 shows the colour variation of the medium; it indicated that higher amount of silver nanoparticles formation occurred at pH 9. Nayak et al. [20] stated that the band at 420 nm indicated the spherical shape of nanoparticles, whereas at 480 nm the particles are different shapes.  Figure 3 shows the XRD pattern of the silver nanoparticles synthesized using* Bacillus brevis*. The XRD pattern indicated strong peaks in the entire spectrum of 2*θ* values ranging from 20 to 80. The silver nanoparticles synthesized in this experiment were in the form of nanocrystals. In the XRD spectrum, the peaks at 2*θ* values of 32.24°, 48.11°, 58.64°, and 77.47° analogous to (111), (200), (220), and (311) planes confirmed the face centered cubic crystalline structure of nanosilver [21]. There was an unassigned peak in the XRD spectrum, due to the presence of bioorganic phase that occurred in the surface of the silver nanoparticles [22].

**Figure 4:**
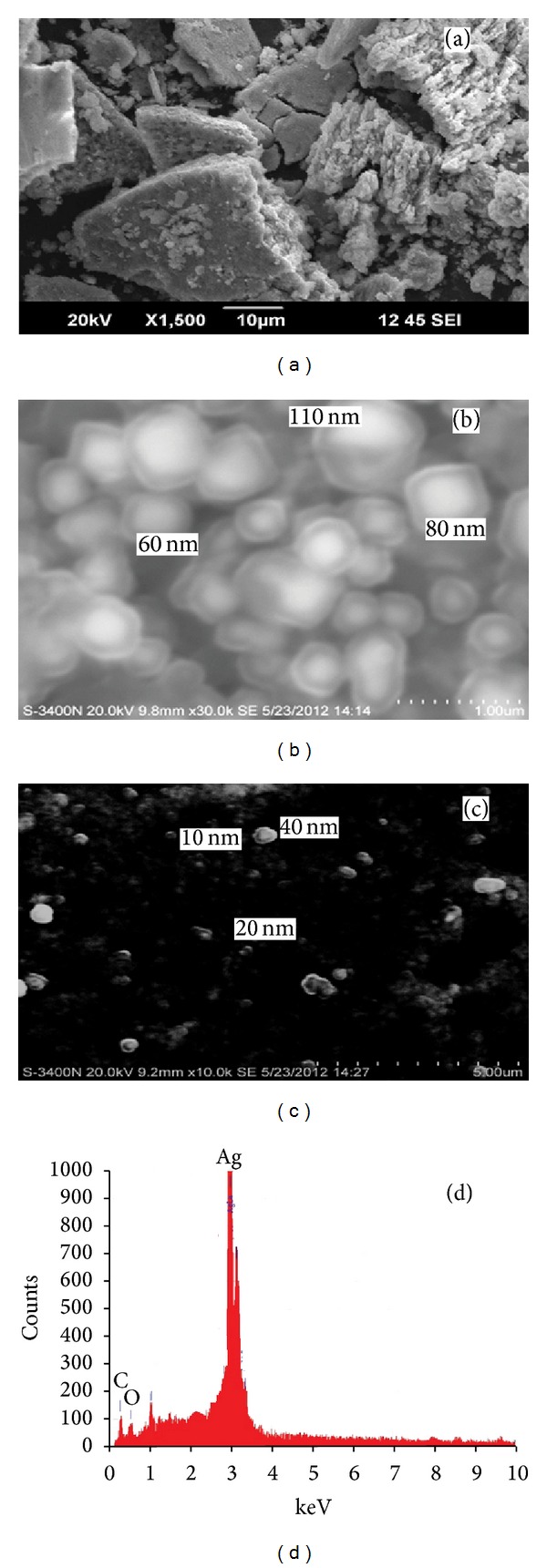
SEM image of the synthesized silver nanoparticles. (a) Original pH (7.1), (b) pH 5, (c) pH 9, (d) EDAX spectrum of silver nanoparticles.

Figures 4(a)–4(d) show the SEM and EDX image of the biosynthesized silver nanoparticles. In Figure 4(a) the particles agglomerated and there was no understandable shape.  Figure 4(b) shows the SEM image of silver nanoparticles synthesized using pH 5; the synthesized particles were hexagonal in shape and the size of the nanoparticles was in the range of 60–110 nm.  Figure 4(c) shows the SEM image of the silver nanoparticles synthesized using pH 9; the particles were spherical and the obtained particles are 10–40 nm in size.  Figure 4(d) shows the EDX spectrum of silver nanoparticles; the strong peak at 3 keV indicated the presence of elemental silver nanoparticles. The size of nanoparticles is high at acidic pH, because the nucleation process for the formation of silver nanocrystal at acidic pH is slow; thus the low amount of large size particles formed. While at high pH, fast nucleation process occurred because of the accessibility of –OH ions; thus high amount of small size particles formed [23].

**Figure 5:**
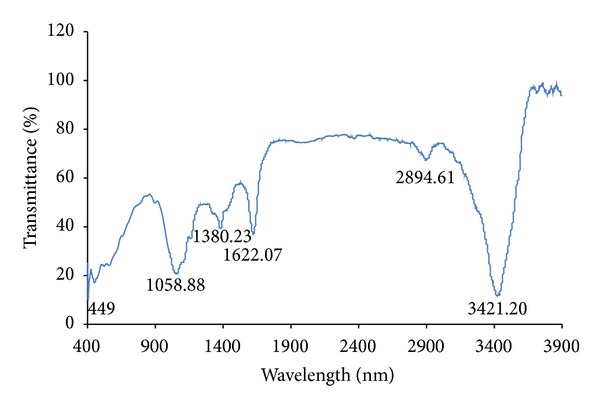
FTIR Spectrum of biosynthesized silver nanoparticles using* Bacillus brevis*.

The FTIR spectrum of silver nanoparticles was synthesized using* Bacillus brevis* (Figure 5). The band at 3412 cm^−1^ and 2918 cm^−1^ represents the O–H, C–C stretching vibration [24]. The band at 1634 cm^−1^ represents the –NH stretching vibration of the amide group [12]. The bands at 1381 cm^−1^ and 1058 cm^−1^ represent the aromatic and aliphatic amines of C–N stretching vibrations of protein [25]. The FTIR results confirmed that the protein might be responsible for the formation of silver nanoparticles [26].

**Figure 6:**
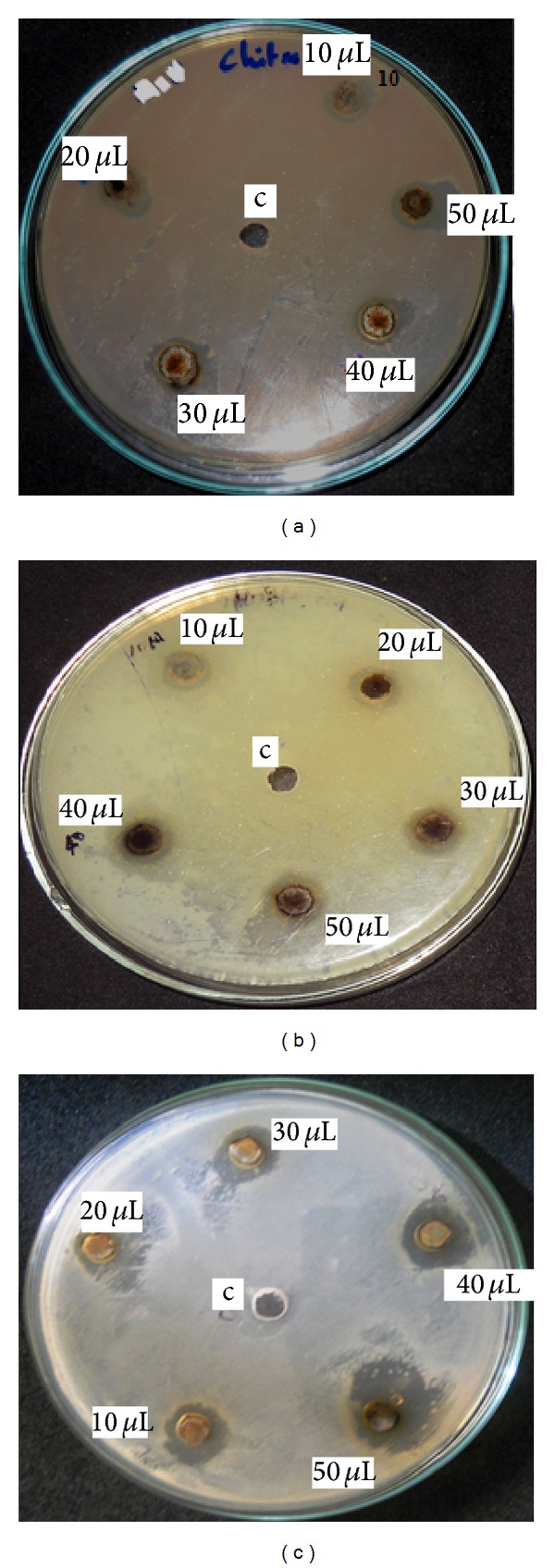
Antibacterial activity of silver nanoparticles. (a) Silver nanoparticles synthesized using original pH of the extracellular aqueous solution, (b) silver nanoparticles synthesized at pH 5, and (c) silver nanoparticles synthesized at pH 9.

The well-diffusion method was used to provide evidence for the antibacterial activity of biosynthesized silver nanoparticles against* E. coli*.  Figure 6 shows the antibacterial effect of silver nanoparticles (synthesized using (a) original pH (7.2) (b) pH-5 and pH-(9)) against* E. coli.* The antibacterial activity of silver nanoparticles was indicated by the formation of the zone and the zone of inhibition measured as mm/diameter. The maximum zone of inhibition occurred at 50 *μ*L concentration of silver nanoparticles. The silver nanoparticles synthesized using pH 9 show higher antibacterial activity (13 mm) against* E. coli*. Silver has been well-known disinfectant for long years. The use of silver compounds is reduced due to some limitations; recently metallic silver in the form of silver nanoparticles shows well antibacterial activity against many microorganisms [27]. Small sized nanoparticles showed more antibacterial activity than large size particles because small sized particles affect a large surface area of the bacteria [28]. There are some possible mechanisms for the antibacterial activity of silver nanoparticles; early studies reported that the electrostatic interaction may be possible reason for the antibacterial activity of silver nanoparticles [29]. Early studies stated that the bacterial proteins are inactivated by the interaction between silver nanoparticles and thiol groups of bacterial protein. Nayak et al. [20] reported the similar results while using silver nanoparticles synthesized by different pH; they stated that the initial pH of the medium, surface area, and shape plays important role in the antibacterial efficiency of silver nanoparticles.

## 4. Conclusion 

The silver nanoparticles were synthesized using* Bacillus brevis* by extracellular method. The different sized and shaped nanoparticles formed while changing the pH of the aqueous solution. The biosynthesized silver nanoparticles were in face centered cubic crystalline structure. The proteins which are present in the bacteria may be possible reason for the synthesis of silver nanoparticles. The pH of the aqueous solution plays important role in the antibacterial activity of silver nanoparticles; the smallest nanoparticles synthesized using pH 9 showed more antibacterial activity than large particles which are synthesized using original pH and pH 5.
